# Assessing the Limitations of Large Language Models in Clinical Practice Guideline–Concordant Treatment Decision-Making on Real-World Data: Retrospective Study

**DOI:** 10.2196/74899

**Published:** 2025-11-03

**Authors:** Tobias Roeschl, Marie Hoffmann, Djawid Hashemi, Felix Rarreck, Nils Hinrichs, Tobias Daniel Trippel, Matthias I Gröschel, Axel Unbehaun, Christoph Klein, Jörg Kempfert, Henryk Dreger, Benjamin O'Brien, Gerhard Hindricks, Felix Balzer, Volkmar Falk, Alexander Meyer

**Affiliations:** 1 Department of Cardiology, Angiology and Intensive Care Medicine, Deutsches Herzzentrum der Charité, Berlin, Germany; 2 Charité – Universitätsmedizin Berlin, corporate member of Freie Universität Berlin and Humboldt-Universität zu Berlin, Charitéplatz 1, Berlin, 10117, Germany, 49 17632864219; 3 Berlin Institute of Health at Charité – Universitätsmedizin Berlin, BIH Biomedical Innovation Academy, BIH Charité Digital Clinician Scientist Program, Berlin, Germany; 4 DZHK (German Centre for Cardiovascular Research), partner site Berlin, Berlin, Germany; 5 Department of Cardiothoracic and Vascular Surgery, Deutsches Herzzentrum der Charité (DHZC), Berlin, Germany; 6 Department of Infectious Diseases and Respiratory Medicine, Charité – Universitätsmedizin Berlin, Berlin, Germany; 7 Department of Cardiac Anesthesiology and Intensive Care Medicine, Deutsches Herzzentrum der Charité (DHZC), Berlin, Germany; 8 Department of Perioperative Medicine, St Bartholomew’s Hospital and Barts Heart Centre, London, United Kingdom; 9 Charité – Universitätsmedizin Berlin, Institute of Medical Informatics, Berlin, Germany; 10 Department of Health Sciences and Technology, Translational Cardiovascular Technologies, Institute of Translational Medicine, Swiss Federal Institute of Technology, Zürich, Switzerland; 11 Berlin Institute for the Foundations of Learning and Data – TU Berlin, Berlin, Germany

**Keywords:** large language models, foundation models, reasoning models, treatment decision-making, aortic stenosis, clinical practice guidelines, medical data processing

## Abstract

**Background:**

Studies have shown that large language models (LLMs) are promising in therapeutic decision-making, with findings comparable to those of medical experts, but these studies used highly curated patient data.

**Objective:**

This study aimed to determine if LLMs can make guideline-concordant treatment decisions based on patient data as typically present in clinical practice (lengthy, unstructured medical text).

**Methods:**

We conducted a retrospective study of 80 patients with severe aortic stenosis who were scheduled for either surgical (SAVR; n=24) or transcatheter aortic valve replacement (TAVR; n=56) by our institutional heart team in 2022. Various LLMs (BioGPT, GPT-3.5, GPT-4, GPT-4 Turbo, GPT-4o, LLaMA-2, Mistral, PaLM 2, and DeepSeek-R1) were queried using either anonymized original medical reports or manually generated case summaries to determine the most guideline-concordant treatment. We measured agreement with the heart team using Cohen κ coefficients, reliability using intraclass correlation coefficients (ICCs), and fairness using the frequency bias index (FBI; FBI >1 indicated bias toward TAVR).

**Results:**

When presented with original medical reports, LLMs showed poor performance (Cohen κ coefficient: −0.47 to 0.22; ICC: 0.0‐1.0; FBI: 0.95‐1.51). The LLMs’ performance improved substantially when case summaries were used as input and additional guideline knowledge was added to the prompt (Cohen κ coefficient: −0.02 to 0.63; ICC: 0.01‐1.0; FBI: 0.46‐1.23). Qualitative analysis revealed instances of hallucinations in all LLMs tested.

**Conclusions:**

Even advanced LLMs require extensively curated input for informed treatment decisions. Unreliable responses, bias, and hallucinations pose significant health risks and highlight the need for caution in applying LLMs to real-world clinical decision-making.

## Introduction

Large language models (LLMs) have recently demonstrated their impressive capabilities in medicine, exemplified by passing medical board exams [1], making correct diagnoses in complex clinical cases [2], and excelling in physician-patient communication [3]. Most recently, the use of LLMs in therapeutic decision-making has been trialed. Several studies have shown that LLMs can make treatment decisions for patients with oncological and cardiovascular diseases that are in substantial agreement with the respective treatment decisions made by clinical experts on tumor boards [4-7] and heart teams (HTs) [8]. However, a common feature of these studies was that the LLMs did not make treatment decisions based on real-world patient data in its original format (eg, discharge letters, imaging reports, etc) but rather made decisions based on preprocessed data.

In clinical practice, relevant patient data, such as patient characteristics, comorbidities, tumor stages, and imaging results, are typically available in free-text format, either as medical text reports or as text entries in the electronic health record, a format that is likely to persist in the near future. In the aforementioned studies, however, decision-relevant patient data were extracted from the original medical reports by the investigators in a preprocessing step before being provided to the LLMs as input in a concise and high-quality form. However, it is still unknown to what extent LLMs can make treatment decisions based on the original medical data, a scenario that could lead to a significant reduction in physician workload and potentially increase guideline adherence and thus improve patient care.

In this study, we investigated the impact of data representation, that is, using original medical reports versus case summaries, on the performance of LLMs in therapeutic decision-making.

As our study population, we selected patients with severe aortic stenosis (AS). This cohort was chosen because the parameters relevant to decision-making are readily quantifiable, the potential for resource optimization is substantial, and the prevalence of the condition is increasing. If left untreated, AS is associated with high morbidity and mortality [9]. Treatment modalities for severe AS include surgical aortic valve replacement (SAVR), transcatheter aortic valve replacement (TAVR), and, to a lesser extent, medical therapy. The choice of the optimal treatment modality depends on several clinical variables, including patient age, estimated surgical risk, comorbidities, and anatomical factors, as specified in the 2021 European Society of Cardiology (ESC) and European Association for Cardio-Thoracic Surgery (EACTS) Guidelines for the management of valvular heart disease [10]. The 2021 ESC/EACTS Guidelines strongly endorse an active, collaborative consultation with a multidisciplinary HT. HTs are comprised of cardiologists, cardiac surgeons, cardiac imaging specialists, and cardiac anesthesiologists. In HT meetings, these experts review a patient’s condition based on patient data laboriously extracted from medical reports before arriving at a treatment decision using a guideline-based approach.

## Methods

### Study Design and Evaluation Framework

**Figure 1.**
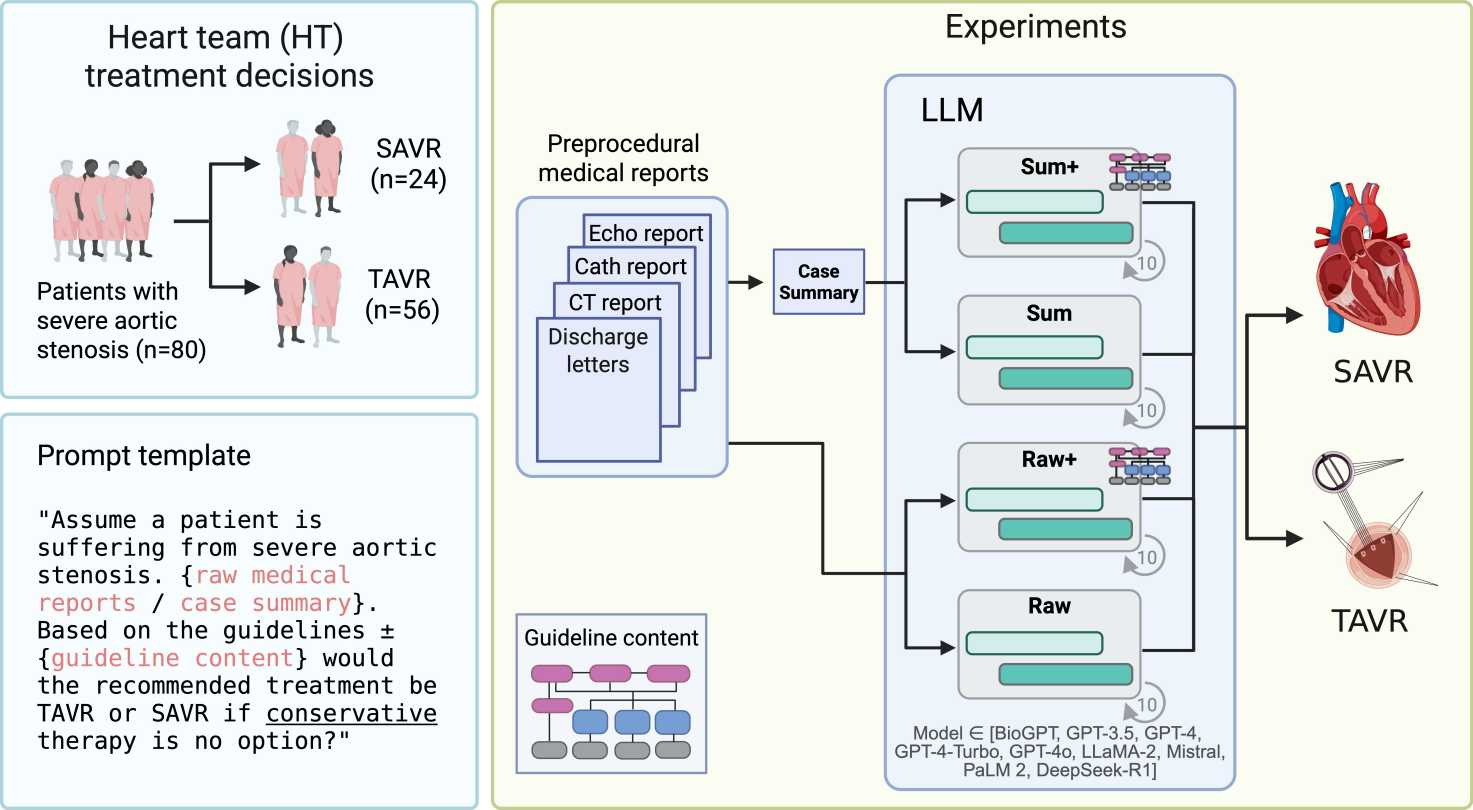
Experimental design. We presented the clinical data of 80 patients with severe aortic stenosis to a large language model (LLM) to receive a treatment decision for either surgical aortic valve replacement (SAVR) or transcatheter aortic valve replacement (TAVR), repeating each query 10 times. To investigate whether injecting guideline knowledge (raw+) into the prompt and/or using case summaries (sum and sum+) instead of the original medical reports (raw) improves LLM performance, we conducted a total of 4 experiments. Case summaries included only decision-relevant patient data and were manually created by physicians. CT: computed tomography.

We presented patient data to an LLM to obtain a treatment decision of either SAVR or TAVR. We assessed the degree of agreement between the treatment decisions provided by the LLM and the treatment decisions provided by the HT. Furthermore, we assessed the decidability, reliability, and fairness of the LLM. Finally, we compared the performance of 7 state-of-the-art LLMs to the performance of a simple non-LLM reference model. In an ablative manner, we studied the effect of using case summaries instead of the original medical reports and adding guideline knowledge to the prompt separately, resulting in 4 distinct experiments (Figure 1).

### Study Population

This study included patients treated at a heart center. We screened all patients with severe degenerative AS who were scheduled for an HT meeting in our hospital information system at 1 campus of our center in 2022. We identified 80 patients with sufficiently digitized documentation. As part of a quaternary care center, our institutional HT receives preselected patients scheduled for invasive AS treatment. Therefore, the number of patients recommended for conservative treatment at our institution is negligible. As a result, we decided to limit the possible therapeutic options for this study to SAVR and TAVR, excluding conservative therapy.

### Ethical Considerations

This study was approved by the research ethics committee of Charité – Universitätsmedizin Berlin (EA1/146/23). The approval included the collection of data based on implied consent owing to the retrospective and observational nature of the study.

### Data Collection

Medical reports were available as PDF files in our hospital information system. For each patient, we included the following preprocedural reports: the 2 most recent discharge letters (including letters from external clinics), invasive coronary angiography report, echocardiography report, computed tomography (CT) scan report, and HT report. We manually anonymized these reports prior to analysis.

HT meeting protocols are standardized documents that contain decision-relevant patient characteristics, such as comorbidities, surgical risk scores, and the final treatment decision of the HT (Multimedia Appendix 1). A detailed description of our institutional HT is provided in Figure S1 in Multimedia Appendix 1.

### LLMs Assessed

The study used several state-of-the-art LLMs, namely GPT-3.5 [11], GPT-4 [12], GPT-4 Turbo, and GPT-4o by OpenAI, and PaLM 2 by Google [13]. In addition, we used the open-source models DeepSeek-R1 [14] by DeepSeek, Mistral-7B [15], LLaMA-2 by Meta [16], and BioGPT [17]. These LLMs had either demonstrated proficiency in similar tasks or had undergone pretraining on medical literature. Model details are provided in Multimedia Appendix 1. The model hyperparameters were set to the default values, except for the temperature, which was set to zero in accordance with previous studies in the medical domain [18]. Temperature is a hyperparameter that controls the randomness of the LLM’s output. Lower values make the output more deterministic and focused, reducing variability and creativity. A detailed description of how we accessed the LLMs and handled input size constraints is given in Multimedia Appendix 1.

### Reference Model

The reference model represented an algorithmic emulation of the 2021 ESC/EACTS Guidelines for the management of valvular heart disease [10]. More specifically, the reference model assigned patients to either SAVR or TAVR according to a flowchart (Figure S2 in Multimedia Appendix 1) and relevant clinical variables (Tables S4 and S5 in Multimedia Appendix 1) [10]. Model details are provided in Table S1 in Multimedia Appendix 1.

### Experiments

Four experiments were conducted to investigate the effects of data preprocessing on LLM performance: raw, raw+, sum, and sum+.

#### Raw

In the raw experiment, we programmatically extracted the text content from the PDF files of relevant medical reports (ie, the 2 most recent discharge letters, invasive coronary angiography report, echocardiography report, and CT scan report) using Tesseract and concatenated these into a unified plain-text file. This text file was then manually anonymized and programmatically inserted into a prompt template. Each prompt included an introductory or continuation phrase and concluded with a request for a treatment decision (Table S6 in Multimedia Appendix 1).

#### Raw+

As it is unknown whether the LLMs we used had sufficient knowledge of clinical practice guidelines (CPGs), we compiled a summary of relevant CPG content from the ESC/EACTS Guidelines [10]. We added this summary to the prompt along with the unified text reports.

#### Sum

To study the effect of content compression, we replaced the original medical reports used in the raw experiment with concise case summaries. These case summaries were created manually by the study team following a predefined template, with each patient characteristic documented in the HT protocol (Figure S1 in Multimedia Appendix 1) either affirmed, negated, or populated with the patient-specific value, as exemplified in Table S6 in Multimedia Appendix 1.

#### Sum+

Case summaries were used as input and were enriched with the CPG summary (Figure 1).

Prompt templates, the CPG summary, and an exemplary case summary are shown in Table S6 in Multimedia Appendix 1.

The LLMs’ responses were manually reviewed and categorized as either “TAVR,” “SAVR,” or “indeterminate.” Indeterminate responses occur when the model output does not match the available answer choices or when the model determines that there is insufficient information to support a decision (Table S7 in Multimedia Appendix 1). To assess reliability and obtain robust estimates of performance metrics, the LLMs were presented with the same prompt input 10 times in succession for each experiment and patient (hereafter referred to as “runs”) to obtain a treatment decision. To prevent memory bias, a new chat session was initiated for each run.

### Performance Metrics

We quantified agreement by means of Cohen κ coefficients. For the sake of completeness, we also calculated accuracies as the proportion of treatment decisions that agreed with those made by the HT; however, we emphasize that due to class imbalance, this metric is only of limited significance and therefore only reported in Table S9 in Multimedia Appendix 1. Decidability was quantified as the proportion of determinate treatment decisions. Bias was quantified using the frequency bias index (FBI), defined as the ratio of predicted to observed treatment decisions for TAVR.

Due to the limitations of individual metrics, we used 2 different metrics to quantify reliability: intraclass correlation coefficients (ICCs) and normalized Shannon entropy. A detailed description of the performance metrics, including strategies for handling indeterminate responses, is provided in Table S8 in Multimedia Appendix 1.

### Statistical Analysis

The characteristics of patients who received SAVR and those who received TAVR were compared using the Student *t*-test for normally distributed continuous variables and the Mann-Whitney *U* test for variables departing from normality. The Shapiro-Wilk test was used to assess normality. The chi-square test was used for binary variables, and the Fisher exact test was used for sparse binary data.

Accuracy and Cohen κ were computed with Python’s sklearn.metrics package (version 1.2.2). ICCs were calculated based on a 1-way random effects, absolute agreement, single-rater model [19] using Python’s pingouin package (version 0.5.3).

## Results

### Patient Characteristics

A total of 80 patients with severe AS who were discussed at our institutional HT in 2022 were included. Of these patients, 24 (30%) underwent SAVR, while 56 (70%) underwent TAVR. Patient characteristics are presented in Table 1.

**Table 1.** Patient characteristics.

Variable	Data availability (%)	Overall (N=80)	SAVR^a^ (n=24)	TAVR^b^ (n=56)	*P* value
Age (years), mean (SD)	100	77.74 (7.5)	70.71 (6.1)	80.75 (5.8)	<.001
Female sex, n (%)	100	36 (45)	8 (33)	28 (50)	.26
Height (cm), mean (SD)	100	168.1 (11.0)	172.5 (11.0)	166.3 (10.6)	.02
Body mass (kg), mean (SD)	100	76.3 (17.0)	79.0 (16.0)	75.1 (17.4)	.35
BMI (kg/m^2^), median (IQR)	100	26.0 (23.0-29.7)	25.9 (23.2-29.0)	26.2 (23.0-29.8)	.66
Logistic EuroSCORE^c^, median (IQR)	31	6.8 (4.5-13.0)	4.5 (2.2-6.8)	8.4 (5.0-16.0)	.20
EuroSCORE II, median (IQR)	99	2.6 (1.6-4.5)	1.8 (1.1-3.1)	2.9 (1.8-4.9)	.02
STS^d^ score, median (IQR)	76	2.8 (1.6-4.5)	1.4 (1.1-3.0)	3.3 (2.1-4.5)	.12
Left ventricular ejection fraction (%), median (IQR)	100	60.0 (54.3-61.3)	60.0 (48.8-62.0)	60.0 (55.0-60.0)	.28
Aortic valve opening area (cm^2^), median (IQR)	100	0.70 (0.60-0.80)	0.80 (0.68-0.80)	0.70 (0.60-0.80)	.18
Arterial hypertension, n (%)	100	59 (74)	18 (75)	41 (73)	>.99
Diabetes mellitus, n (%)	100	22 (28)	6 (25)	16 (29)	.96
Hyperlipidemia, n (%)	100	51 (64)	13 (54)	38 (68)	.36
Previous cardiac surgery, n (%)	100	1 (1)	0 (0)	1 (2)	>.99
Frailty, n (%)	100	7 (9)	0 (0)	7 (13)	.17
Sequelae of chest radiation, n (%)	100	0 (0)	0 (0)	0 (0)	>.99
Porcelain aorta, n (%)	100	0 (0)	0 (0)	0 (0)	>.99
Expected patient-prosthesis mismatch, n (%)	100	1 (1)	0 (0)	1 (2)	>.99
Severe chest deformation or scoliosis, n (%)	100	7 (9)	1 (4)	6 (11)	.60
Severe coronary artery disease requiring revascularization, n (%)	100	6 (8)	5 (21)	1 (2)	.01
Left ventricular ejection fraction ≤40%, n (%)	100	6 (8)	3 (13)	3 (5)	.52
Active neoplasia, n (%)	100	7 (9)	2 (8)	5 (9)	>.99
Liver cirrhosis, n (%)	100	1 (1)	0 (0)	1 (2)	>.99
Chronic obstructive pulmonary disease (GOLD^e^ stage ≥3), n (%)	100	5 (6)	1 (4)	4 (7)	>.99
Pulmonary arterial hypertension, n (%)	100	8 (10)	3 (13)	5 (9)	.94
Under immunosuppressive therapy, n (%)	100	10 (13)	2 (8)	8 (14)	.71

a SAVR: surgical aortic valve replacement.

b TAVR: transcatheter aortic valve replacement.

c EuroSCORE: European System for Cardiac Operative Risk Evaluation.

d STS: Society of Thoracic Surgeons.

e GOLD: Global Initiative for Chronic Obstructive Lung Disease.

### Qualitative Analysis

The LLMs’ outputs ranged from nonsensical treatment recommendations (eg, heart transplant) and purely fabricated content to correctly assessing the patient’s status, choosing the correct treatment option, and supporting the treatment decision with additional anatomical insights (Table 2). Qualitative analysis revealed that smaller models (eg, BioGPT) tended to provide conflicting treatment recommendations for the same patient. In contrast, the frontier models (eg, GPT-4 and PaLM 2) consistently provided the same treatment recommendation when presented with the same patient data repeatedly over 10 runs.

In each experiment, all LLMs produced hallucinations of varying severity and frequency. These included instructional, contextual, and factual inconsistencies (Table 2).

**Table 2.** Representative responses from the LLMs^a^.

Model	Experiment	Patient characteristics	LLM response^b^^,^^c^	HT^d^ treatment decision	Interpretation
PaLM 2	Raw	56-year-old male; EuroSCORE^e^ II: 0.55%; no comorbidities except diffuse, mild coronary atherosclerosis and arterial hypertension; no relevant anatomical aspects to consider	Response: “Based on the provided information, the recommended treatment for the patient would be transcatheter aortic valve replacement (TAVR). The patient has severe aortic stenosis, which is causing symptoms and has been confirmed by echocardiography and cardiac catheterization. The patient is also *at high risk for surgical aortic valve replacement (SAVR) due to their age, comorbidities, and the presence of diffuse coronary artery disease*^f^. TAVR is a less invasive procedure than SAVR and is associated with a *lower risk of complications*^f^. Therefore, *TAVR*^f^ would be the preferred treatment option for this patient.”	SAVR^g^	The LLM incorrectly recommended TAVR^h^ based on falsely extracted and/or interpreted patient characteristics (factual inconsistency). According to the literature, it has not yet been conclusively determined whether TAVR is associated with fewer complications than SAVR [20] (factual inconsistency/bias).
BioGPT	Raw	69-year-old male; EuroSCORE II: 7.2%; postcardiac arrest syndrome, frailty, long-term mechanical ventilation, and liver cirrhosis; no relevant anatomical aspects to consider	Response: “*Surgical replacement of the aortic valve. The patient was referred to the operating room for AVR. The operation was performed under general anesthesia and extracorporeal circulation ... The operation was performed with a median sternotomy. The ascending aorta was dissected free and opened…and the coronary arteries were visualized…*^f^”	TAVR	The LLM falsely stated a surgery report from a procedure this patient never received and that was not mentioned in the prompt (context inconsistency).
BioGPT	Raw	75-year-old female; EuroSCORE II: 2.4%; STS^i^ score: 2.9%; COPD^j^ (GOLD^k^ Stage III); pulmonary hypertension and frailty; no relevant anatomical aspects to consider	Response: “*Heart transplantation. - The patient’s family decided against surgery. - The patient died due to heart failure.*^f^”	TAVR	The LLM did not follow the instruction to choose between SAVR and TAVR but opted for heart transplantation (instruction inconsistency). The LLM fabricated a clinical course that was not mentioned in the prompt (context inconsistency).
GPT-3.5	Sum	72-year-old female; EuroSCORE II: 1.6%; STS score: 1.1%; no relevant comorbidities; no relevant anatomical aspects to consider	Response_1_: “Based on the given information, the recommended treatment for a person with severe aortic valve stenosis and no other significant comorbidities would be *SAVR*^l^ (Surgical Aortic Valve Replacement) if conservative therapy is not an option.” Response_2_: “Based on the provided information, the recommended treatment for a person with severe aortic valve stenosis, who does not have any contraindications or relevant comorbidities, would be *TAVI*^f^ (Transcatheter Aortic Valve Implantation) rather than SAVR (Surgical Aortic Valve Replacement).”	SAVR	The LLM gave opposite answers to the exact same question (unreliability). The LLM incorrectly recommended TAVR based on the absence of comorbidities (factual inconsistency).
PaLM 2	Raw+	56-years-old male; EuroSCORE II: 0.55%; STS score: 0.7%; no comorbidities except arterial hypertension; no relevant anatomical aspects to consider	Response: “Based on the guidelines, the recommended treatment would be transcatheter aortic valve replacement (*TAVR*^f^). The patient is 56 years old and at *high surgical risk*^f^ (STS-PROM score >8% or EuroSCORE II >8 %) due to *severe frailty*^f^ and *previous cardiac surgery*^f^. TAVR is the preferred treatment for patients who are inoperable or at high surgical risk.”	SAVR	The LLM falsely claimed that the patient is at high surgical risk due to frailty and previous cardiac surgery, which are both entirely fabricated (factual inconsistency).
GPT-3.5	Sum+	81-year-old female; logistic EuroSCORE: 8.44%; EuroSCORE II: 1.82%; STS score: 4.33%; stage 3A chronic kidney disease; no relevant anatomical aspects to consider	Response: “Based on the guidelines provided, the recommended treatment for the patient would be *SAVR*^f^ (surgical aortic valve replacement) if conservative therapy is not an option. This is because the patient is 81 years old and falls into the *older age category*^l^, and *their logistic EuroSCORE*^f^ is 8.44%, which is above the threshold for TAVR (transcatheter aortic valve replacement) recommendation.”	TAVR	The LLM incorrectly mentioned an association between the logistic EuroSCORE and surgical risk prohibiting SAVR (context inconsistency). The LLM correctly assigned the patient to the older age category but then incorrectly recommended SAVR as the optimal therapy (logical inconsistency).
GPT-4	Raw	65-year-old female; EuroSCORE II: 2.5%; STS score: 1.4%; no relevant comorbidities; ascending aortic aneurysm (48 mm) mentioned in the CT^m^ scan report	Response: “Based on the guidelines, the recommended treatment would be Surgical Aortic Valve Replacement (*SAVR*^l^) as the patient has a severe aortic stenosis and an *ascending aortic aneurysm*^l^. Transcatheter Aortic Valve Implantation (TAVI) is typically recommended for patients who are at *high or prohibitive surgical risk, which does not seem to be the case here.*^l^”	SAVR	Correct treatment decision plus additional extraction of decision-relevant clinical data.
LLaMA-2	Sum+	68-year-old male; EuroSCORE II: 1.29%; STS score: 3.04%; COPD; no relevant anatomical aspects to consider	Reponse_1_: “Based on the guidelines provided, the recommended treatment for this patient would be TAVI. The patient’s age (68 y old) and *low surgical risk*^l^ (EuroSCORE II 1.29 %, STS score 3.037 %) make them a suitable candidate for *TAVI*. Additionally, the patient does not have any *contraindications for TAVI*^f^*, such as severe frailty, previous cardiac surgery, or significant thorax deformity.*^f^” Response_2_: ‘Based on the information provided, the patient’s EuroSCORE II is 1.29% and STS score is 3.037 %, which indicates that they are at *low surgical risk*^l^. The patient is also *younger than 75 years*^l^ old. Therefore, according to the guidelines, *SAVR*^l^ is the recommended treatment option for this patient.”	SAVR	The LLM correctly identified low surgical risk but then incorrectly rejected SAVR as a therapeutic option (logical inconsistency). The LLM incorrectly mentioned factors favoring TAVR from the prompt (context inconsistency). Although the LLM correctly selected SAVR the second time, it gave the opposite response to the exact same question (unreliability).
DeepSeek-R1	Raw+	65-year-old male; EuroSCORE II: 0.92%; STS score: 0.73%; end-stage renal disease requiring hemodialysis; no relevant anatomical aspects to consider	Response: “… Based on the 2021 ESC/EACTS guidelines and the patient’s clinical profile, the recommended treatment would be *TAVR*^f^. Reasoning: Patient age (65 y) is below 75 but has significant comorbidities, *including terminal renal insufficiency, indicating high surgical risk*^f^. Suitable tricuspid aortic valve anatomy for TAVR. High surgical risk factors favor TAVR over SAVR.”	SAVR	The LLM overestimated the effect of end-stage renal disease on surgical risk, which was still relatively low overall, as evidenced by low surgical risk scores (factual inconsistency).

a LLM: large language model.

b The LLMs’ treatment responses included well-informed decisions but also hallucinations ranging from obvious misinformation to absurd treatment recommendations and logical errors. We largely adhered to the taxonomy for the description of hallucinations established by Huang et al [21].

c LLM responses with subscripts indicate responses to the same question (obtained during 10 runs).

d HT: heart team.

e EuroSCORE: European System for Cardiac Operative Risk Evaluation.

f The italicized part indicates an incorrect or harmful response.

g SAVR: surgical aortic valve replacement.

h TAVR: transcatheter aortic valve replacement.

i STS: Society of Thoracic Surgeons.

j COPD: chronic obstructive pulmonary disease.

k GOLD: Global Initiative for Chronic Obstructive Lung Disease.

l The italicized part indicates a correct or useful response.

m CT: computed tomography.

### Quantitative Analysis

**Figure 2.**
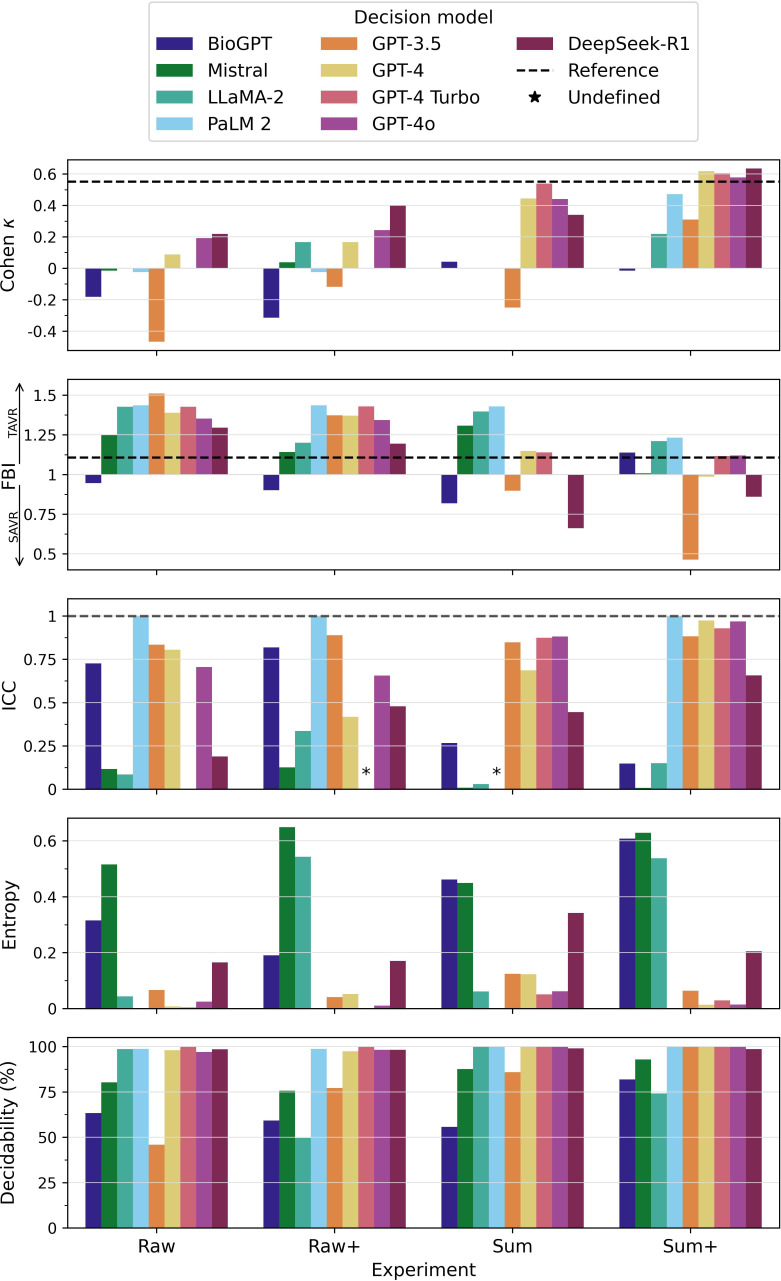
Performance metrics of the large language models are shown for the 4 experiments conducted. The dashed line represents the reference model. Cohen κ coefficients ≤0 indicate no agreement, 0.01‐0.20 indicate slight agreement, 0.21‐0.40 indicate fair agreement, 0.41‐0.60 indicate moderate agreement, 0.61‐0.80 indicate substantial agreement, and 0.81‐1.0 indicate almost perfect agreement [20] with the heart team’s treatment decisions. Frequency bias index (FBI) values >1 indicate bias toward transcatheter aortic valve replacement (TAVR) and <1 indicate bias toward surgical aortic valve replacement (SAVR). Intraclass correlation coefficients (ICCs) <0.5 indicate poor test-retest reliability, 0.50‐0.75 indicate moderate reliability, 0.75‐0.90 indicate good reliability, and >0.90 indicate excellent reliability [19]. Instances where ICCs were undefined are marked by asterisks. Entropy values close to 0 indicate low output variation, and entropy values close to 1 indicate high output variation. Decidability was defined as the proportion of nonindeterminate treatment decisions. The exact numerical values for the performance metrics are displayed in Table S9 in Multimedia Appendix 1.

Figure 2 and Table S9 in Multimedia Appendix 1 present the performance metrics. In the raw experiment, LLMs’ treatment decisions were in poor agreement with the HT. In this experiment, DeepSeek-R1 showed the highest agreement with the HT, with a Cohen κ coefficient of 0.22. Some LLMs gave indeterminate treatment recommendations in up to 54% of cases (eg, GPT-3.5) and showed low reliability as evidenced by low ICCs and high entropy values (eg, Mistral, LLaMA-2, and DeepSeek-R1). FBIs were substantially higher than 1.0 for all LLMs, except BioGPT, indicating a bias toward TAVR. The reference model outperformed the LLMs in the raw experiment regarding the metrics we assessed.

In the raw+ experiment, DeepSeek-R1 again showed the highest agreement with the HT with a Cohen κ coefficient of 0.40, indicating fair agreement. The performance metrics of the other LLMs did not change substantially in the raw+ experiment. However, the performance metrics of most LLMs substantially improved in the sum experiment and peaked in the sum+ experiment, where some LLMs (eg, GPT-4 models and DeepSeek-R1) drew level with the reference model.

A general trend toward more concordant treatment decisions, fewer indeterminate responses, increased reliability, and less bias toward TAVR was observed with increasing data preprocessing and information enrichment efforts from the raw experiment to the sum+ experiment (Figures2  3).

**Figure 3.**
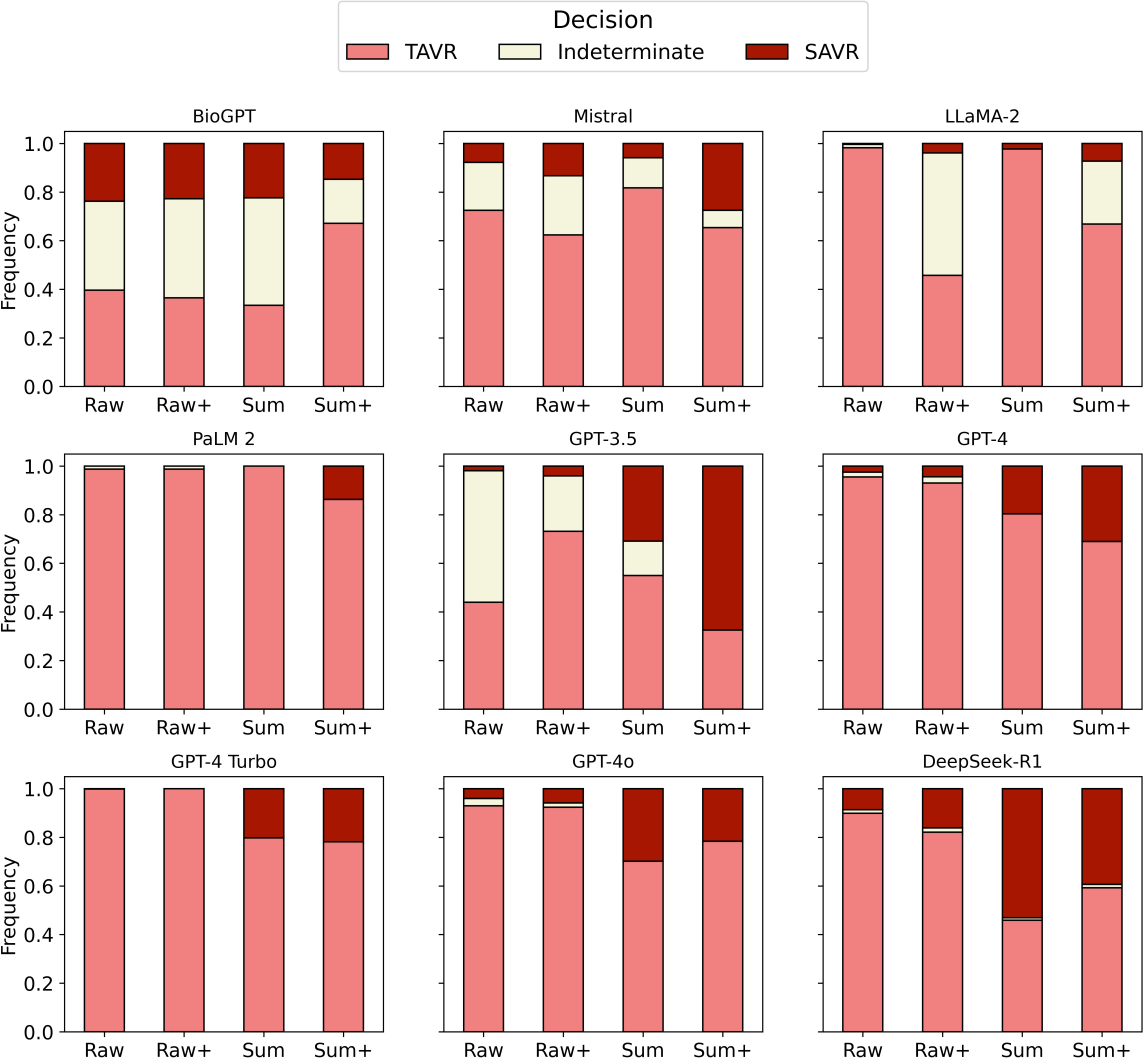
Frequencies of the treatment decisions of the large language models in the 4 experiments conducted. A general trend toward increasing decidability and an increasing proportion of treatment decisions favoring surgical aortic valve replacement (SAVR) could be observed between the raw experiment and the sum+ experiment. TAVR: transcatheter aortic valve replacement.

## Discussion

### LLM Performance With Original Clinical Data

To our knowledge, this is the first study to evaluate the impact of input data representation, including real-world medical data, on the ability of LLMs to make guideline-concordant treatment decisions.

### Current LLMs Make Incorrect Decisions Based on Original Clinical Data

Our analysis revealed that LLMs struggled to process original medical reports effectively, often outputting “TAVR” or providing indeterminate responses. The LLMs showed low agreement with the HT, exhibited undecidability and unreliability, and displayed a strong bias toward TAVR. The considerably high accuracies (Table S9 in Multimedia Appendix 1) observed with some LLMs in the raw experiment can be largely attributed to the class imbalance within our patient cohort, where 70% of patients received TAVR.

### LLMs Require Extensive Data Preprocessing to Make Sound Therapeutic Decisions

Performance improved substantially when physician-made case summaries were used as input and when guideline knowledge was added to the prompts. The GPT-4 models and DeepSeek-R1 stood out as the most capable LLMs in our experiments. When given case summaries and a CPG summary, these 2 models showed substantial agreement with HT and drew level with the reference model in terms of interrater agreement, decidability, and bias.

### Data Representation Affects LLM Performance

GPT-4o, a distilled and streamlined version of GPT-4, and DeepSeek-R1, a model with enhanced reasoning abilities, showed more promising results than previous-generation LLMs when confronted with real-world medical data (raw and raw+ experiments); however, their performances remain insufficient for clinical application. The fact that even state-of-the-art LLMs show significant stochastic variations in decision-making, and thus unreliability, further supports this finding.

An explanation for the underperformance of LLMs in the raw experiment is not immediately apparent due to their opaque nature and a lack of established tools that allow the direct examination of input-output correlations. However, the underperformance cannot be attributed to a lack of guideline knowledge or incorrectly applied guideline knowledge since the performance in the raw+ experiment was, in general, similar to that in the raw experiment and since LLMs can presumably apply clinical knowledge to clinical cases as shown in their ability to pass medical board exams [1,22].

This, along with the significant performance gains observed when providing case summaries instead of original medical reports, suggests that input data representation is the most critical factor in LLM performance. This finding is consistent with the fact that virtually all studies, which showed that LLMs make sound treatment decisions, used preprocessed clinical data as input [4-8]. Of note is the study by Salihu et al [8]. In this study, data from patients with severe AS were provided to GPT-4 to obtain a treatment decision for either TAVR, SAVR, or conservative management. Patient data were provided in the form of a standardized multiple-choice questionnaire with 14 key clinical variables as input, similar to our sum experiments. GPT-4 treatment decisions were in substantial agreement with HT treatment decisions, a finding that we were able to reproduce in our experiments. Similarly, in studies on tasks beyond therapeutic decision-making, such as answering board exam questions [1,23] and diagnosing complex clinical cases [2,24,25], LLMs performed particularly well when the input data were concise and information-dense.

Basic research has indicated that LLMs struggle with lengthy texts [26] spanning over multiple prompts, potentially leading to memory loss [27] and texts with a low signal-to-noise ratio [28]. A study by Levy et al [29] demonstrated that LLM reasoning performance declined notably with increasing input length. Specifically, the authors observed a 26% drop in LLM performance when the input length was artificially increased from 250 to 3000 tokens, that is, a range of input lengths comparable to that in our study (Table S3 in Multimedia Appendix 1).

Recently, Hager et al [30] investigated the ability of LLMs to correctly diagnose patients presenting to the emergency department with abdominal pain. In this study, it was shown that deliberately withholding relevant clinical information from the LLMs paradoxically improved their diagnostic accuracy. Overall, this implies that LLMs are sensitive to both the signal-to-noise ratio and the sheer quantity of information provided.

### LLMs Are Not Yet Ready for Clinical Decision-Making

The results obtained with preprocessed patient data in our study and in previous studies demonstrate the potential of LLMs in medicine. However, the use of curated and preprocessed data does not reflect the clinical situation: To this day, the communication of clinical data within hospitals is largely based on unstructured free text.

Health care professionals have high expectations of artificial intelligence (AI) to reduce their workload. This is not the case when physicians must manually extract and prepare key patient data for LLMs, as data extraction, not the actual decision-making task, is usually the most labor-intensive step.

Once key patient data have been extracted and prepared as input, simpler machine learning models (eg, tree-based models) could be used alternatively to provide decision support. In our study, as well as in the study by Salihu et al [8], simple reference models performed comparably to GPT-4, suggesting that non-LLM models could outperform LLMs if trained appropriately. In addition, nongenerative models do not exhibit undesirable behaviors, such as hallucinations and unreliability [21,31,32], and provide explainability and established measures of uncertainty quantification, which are 2 hallmarks of reasonable AI [33] that are currently not adequately implemented for LLMs [34-36].

Another hallmark of reasonable AI is to address algorithmic bias [37]. It is conceivable that the bias we observed in virtually all LLMs in our study could be due to LLMs being exposed to an abundance of TAVR-related internet literature during training compared to SAVR, subsequently influencing the treatment decisions.

A reasonable approach could be to use LLMs to extract clinical data [38] and generate input for downstream deterministic models, which then perform the decision-making. While this strategy should ideally exploit the strengths of LLMs and well-established machine learning classifiers, its effectiveness remains to be proven in future studies.

### Limitations

Our study has some limitations, including a small patient cohort from a single center and the retrospective nature of our investigation. Nevertheless, the size of our study cohort (n=80) was comparable to previous key publications [2,39] studying the performance of LLMs in medicine, and we assume that our patient cohort was sufficiently large given the clear trends we observed.

The HT decisions against which we compared the LLMs’ treatment decisions may themselves be nonobjective and deviate from the CPGs. We manually reviewed the HT treatment decisions and found no substantial deviations from the CPGs. Since treatment decisions are ultimately made by a team of physicians (ie, human individuals), the ground truth in experiments such as ours is inherently susceptible to some degree of subjectivity.

Given the limited cohort size and the considerable length of the medical reports, few-shot prompting or fine-tuning was not a viable option. We did not employ more sophisticated prompting techniques, such as chain-of-thought [40], and confined hyperparameter tuning to the temperature parameter. Moreover, given the rapid pace of LLM development, it is plausible that the most recently released reasoning-focused models (eg, GPT-o3 and Grok 4) may outperform those evaluated in our study. Accordingly, our findings should be interpreted as a reflection of the current state of model performance.

The majority of LLMs evaluated were primarily trained on English-language data. While recent studies suggest that newer models exhibit greater language agnosticism, it remains plausible that our use of German-language clinical reports contributed to reduced model performance, thereby limiting the generalizability of our findings to other languages and clinical settings [41].

We acknowledge that the off-the-shelf LLMs used in our study may exhibit biases due to the underrepresentation of certain ethnic, gender, or socioeconomic groups in their training data. However, given the limited size of our cohort, we were not able to systematically assess or stratify model performance across these dimensions.

Lastly, we did not investigate whether incorporating imaging data as additional input for multimodal LLMs, such as GPT-4o, could have improved model performance in our task. While this is theoretically plausible, recent research suggests that the effectiveness of multimodal models in clinical applications depends heavily on the quality of the accompanying textual context [42,43]. Given that relevant imaging findings were generally described in detail in the imaging reports, we assume that the inclusion of imaging data in our specific use case would likely have had only a limited impact on overall model performance.

### Conclusions

Our experiments are among the most challenging tasks LLMs have been asked to perform in the medical sciences. Overall, we conclude that LLMs are currently not suitable as decision makers for the treatment of patients with severe AS, as our results suggest that LLMs require elaborate preprocessing of patient data to make guideline-concordant treatment decisions. Thus, we do not share the medical community’s concern that staff will be replaced by AI [44] in clinical decision-making in the near future.

Our findings suggest that LLMs should be used cautiously, particularly by medical laypersons seeking medical advice, such as second opinions. Users without extensive domain knowledge may receive treatment recommendations at a level similar to our raw experiments. This is because medical laypersons may not be able to support prompts with guideline knowledge or create case summaries of sufficient quality but will only be able to use original medical reports. The findings in the study by Hager et al [30] suggest that LLMs perform poorly when collecting additional patient data sequentially, as physicians would during a patient-physician dialogue. This suggests that the alternative to our approach—not providing all clinical data to the LLM at once, but having medical laypersons provide essential information incrementally during a chat session—is also likely to lead to suboptimal therapeutic recommendations.

Finally, medical laypersons may not be able to recognize hallucinations as effectively as medical professionals. This, combined with the eloquent and persuasive linguistic style of most LLMs, has the potential to mislead users by creating an illusion of greater certainty than warranted, aggravating the hazardous effects of incorrect treatment recommendations.

## Supplementary material

10.2196/74899 Multimedia Appendix 1 Additional information to support the study findings.

## Abbreviations

AI: artificial intelligence
AS: aortic stenosis
CPG: clinical practice guideline
CT: computed tomography
EACTS: European Association for Cardio-Thoracic Surgery
ESC: European Society of Cardiology
FBI: frequency bias index
HT: heart team
ICC: intraclass correlation coefficient
LLM: large language model
SAVR: surgical aortic valve replacement
TAVR: transcatheter aortic valve replacement

## References

[1] Kung TH, Cheatham M, Medenilla A (2023). Performance of ChatGPT on USMLE: potential for AI-assisted medical education using large language models. PLOS Digit Health.

[2] Kanjee Z, Crowe B, Rodman A (2023). Accuracy of a generative artificial intelligence model in a complex diagnostic challenge. JAMA.

[3] Tu T, Palepu A, Schaekermann M (2024). Towards conversational diagnostic AI. arXiv.

[4] Sorin V, Klang E, Sklair-Levy M (2023). Large language model (ChatGPT) as a support tool for breast tumor board. NPJ Breast Cancer.

[5] Aghamaliyev U, Karimbayli J, Giessen-Jung C (2024). ChatGPT’s gastrointestinal tumor board tango: a limping dance partner?. Eur J Cancer.

[6] Kozel G, Gurses ME, Gecici NN (2024). Chat-GPT on brain tumors: an examination of artificial intelligence/machine learning’s ability to provide diagnoses and treatment plans for example neuro-oncology cases. Clin Neurol Neurosurg.

[7] Lukac S, Dayan D, Fink V (2023). Evaluating ChatGPT as an adjunct for the multidisciplinary tumor board decision-making in primary breast cancer cases. Arch Gynecol Obstet.

[8] Salihu A, Meier D, Noirclerc N (2024). A study of ChatGPT in facilitating heart team decisions on severe aortic stenosis. EuroIntervention.

[9] Roth GA, Mensah GA, Johnson CO (2020). Global burden of cardiovascular diseases and risk factors, 1990-2019: update from the GBD 2019 study. J Am Coll Cardiol.

[10] Vahanian A, Beyersdorf F, Praz F (2022). 2021 ESC/EACTS Guidelines for the management of valvular heart disease. Eur Heart J.

[11] Ye J, Chen X, Xu N (2023). A comprehensive capability analysis of GPT-3 and GPT-3.5 series models. arXiv.

[12] Achiam J, Adler S, Agarwal S (2024). GPT-4 technical report. arXiv.

[13] Anil R, Dai AM, Firat O (2023). PaLM 2 technical report. arXiv.

[14] Guo D, Yang D, Zhang H (2025). DeepSeek-R1: incentivizing reasoning capability in LLMs via reinforcement learning. arXiv.

[15] Jiang AQ, Sablayrolles A, Mensch A (2023). Mistral 7B. arXiv.

[16] Touvron H, Martin L, Stone K (2023). Llama 2: open foundation and fine-tuned chat models. arXiv.

[17] Luo R, Sun L, Xia Y (2022). BioGPT: generative pre-trained transformer for biomedical text generation and mining. Brief Bioinformatics.

[18] Liévin V, Hother CE, Motzfeldt AG, Winther O (2022). Can large language models reason about medical questions?. arXiv.

[19] Koo TK, Li MY (2016). A guideline of selecting and reporting intraclass correlation coefficients for reliability research. J Chiropr Med.

[20] Virtanen MPO, Eskola M, Jalava MP (2019). Comparison of outcomes after transcatheter aortic valve replacement vs surgical aortic valve replacement among patients with aortic stenosis at low operative risk. JAMA Netw Open.

[21] Huang L, Yu W, Ma W (2024). A survey on hallucination in large language models: principles, taxonomy, challenges, and open questions. arXiv.

[22] Cai Y, Wang L, Wang Y (2023). MedBench: a large-scale chinese benchmark for evaluating medical large language models. arXiv.

[23] Gilson A, Safranek CW, Huang T (2023). How does ChatGPT perform on the United States Medical Licensing Examination (USMLE)? The implications of large language models for medical education and knowledge assessment. JMIR Med Educ.

[24] Eriksen AV, Möller S, Ryg J (2024). Use of GPT-4 to diagnose complex clinical cases. NEJM AI.

[25] Novak A, Zeljković I, Rode F (2024). The pulse of artificial intelligence in cardiology: a comprehensive evaluation of state-of-the-art large language models for potential use in clinical cardiology. medRxiv.

[26] Liu NF, Lin K, Hewitt J (2023). Lost in the middle: how language models use long contexts. arXiv.

[27] Sejnowski TJ (2023). Large language models and the reverse turing test. Neural Comput.

[28] Wang B, Wei C, Liu Z, Lin G, Chen NF (2024). Resilience of large language models for noisy instructions. arXiv.

[29] Levy M, Jacoby A, Goldberg Y (2024). Same task, more tokens: the impact of input length on the reasoning performance of large language models. arXiv.

[30] Hager P, Jungmann F, Holland R (2024). Evaluation and mitigation of the limitations of large language models in clinical decision-making. Nat Med.

[31] Singhal K, Azizi S, Tu T (2023). Large language models encode clinical knowledge. Nature New Biol.

[32] Roustan D, Bastardot F (2025). The clinicians’ guide to large language models: a general perspective with a focus on hallucinations. Interact J Med Res.

[33] Sivarajah U, Wang Y, Olya H, Mathew S (2023). Responsible artificial intelligence (AI) for digital health and medical analytics. Inf Syst Front.

[34] Luo H, Specia L (2024). From understanding to utilization: a survey on explainability for large language models. arXiv.

[35] Liu L, Pan Y, Li X, Chen G (2024). Uncertainty estimation and quantification for LLMs: a simple supervised approach. arXiv.

[36] Quttainah M, Mishra V, Madakam S, Lurie Y, Mark S (2024). Cost, usability, credibility, fairness, accountability, transparency, and explainability framework for safe and effective large language models in medical education: narrative review and qualitative study. JMIR AI.

[37] Kim J, Vajravelu BN (2025). Assessing the current limitations of large language models in advancing health care education. JMIR Form Res.

[38] Dagdelen J, Dunn A, Lee S (2024). Structured information extraction from scientific text with large language models. Nat Commun.

[39] Huang AS, Hirabayashi K, Barna L, Parikh D, Pasquale LR (2024). Assessment of a large language model’s responses to questions and cases about glaucoma and retina management. JAMA Ophthalmol.

[40] Wei J, Wang X, Schuurmans D Chain-of-thought prompting elicits reasoning in large language models.

[41] Rosoł M, Gąsior JS, Łaba J, Korzeniewski K, Młyńczak M (2023). Evaluation of the performance of GPT-3.5 and GPT-4 on the Polish Medical Final Examination. Sci Rep.

[42] Günay S, Öztürk A, Özerol H, Yiğit Y, Erenler AK (2024). Comparison of emergency medicine specialist, cardiologist, and chat-GPT in electrocardiography assessment. Am J Emerg Med.

[43] Zeljkovic I, Novak A, Lisicic A (2025). Beyond text: the impact of clinical context on GPT-4’s 12-lead electrocardiogram interpretation accuracy. Can J Cardiol.

[44] Fogo AB, Kronbichler A, Bajema IM (2024). AI’s threat to the medical profession. JAMA.

