# Supplementary material for: Assessing the Limitations of Large Language Models in Clinical Practice Guideline–Concordant Treatment Decision-Making on Real-World Data: Retrospective Study
Source: JMIRx Med. 2025 Nov 3;6:e74899. doi: 10.2196/74899 (PMC12587749; doi:10.2196/74899)
Supplement: Multimedia Appendix 1 [file xmed-v6-e74899-s001.docx]

**Multimedia Appendix 1.** Additional information to support the study findings.

## Supplementary methods

### Access to LLMs

We accessed the GPT models and PaLM 2 using the application programming interfaces (API) of OpenAI and Google, respectively. Mistral, DeepSeek-R1 and Llama 2 were accessed through the REST API of perplexity. BioGPT was downloaded from Huggingface and run locally.^1^

For all LLMs except BioGPT and Mistral, we were able to clearly demonstrate that the LLMs were familiar with the 2021 ESC/EACTS Guidelines for the management of valvular heart disease^2^ and could correctly recite key contents.

### Language

All medical reports were originally available in German and were fed into the LLMs as such except for BioGPT. Since BioGPT has not been sufficiently trained on German text data, we translated the original medical reports to English using Python’s deep-translator (version 1.11, module: GoogleTranslator) for these experiments.

### Handling of input size constraints

When the model-specific text input sizes were exceeded, we broke up the text into chunks and modified the prompt to inform the model about contiguous patient data. We established context by adding the preceding output. The context sizes are given in Table S1 and range from 1,024 to 128,000 tokens. The median token counts and average number of prompts are shown in Table S2 and Table S3.

### Institutional Heart Team

Our institutional Heart Team (HT) is comprised of interventional cardiologists, cardiac surgeons, imaging specialists and cardiac anesthesiologists. It is mandatory that at least one representative of the aforementioned specialties participates in an HT meeting. HT meetings are held every two weeks to make treatment decisions for patients with coronary artery disease, valve disorders and structural heart disease.

The HT follows a structured decision-making process outlined in the HT protocol (Figure S1). In this process, decision-relevant patient data (e.g., patient characteristics, comorbidities, anatomical aspects) previously extracted from discharge letters and diagnostic imaging reports is presented along with imaging scans to the HT members. Surgical risk scores are calculated and documented in the HT protocol along with decision-relevant patient data. Patients are assigned to the respective treatment modality according to a guideline-guided approach as depicted by the structure of the HT protocol.

For patients in our study cohort, the ESC/EACTS Guidelines for the management of valvular heart disease^2^, published online in August 2021, were used as the basis for decision-making. The members of the Heart Team are collectively committed to follow these guidelines. If a patient requests a different therapy from that recommended by the Heart Team, this is documented in the HT protocol. In our study, the therapy decisions made by the LLMs were compared to the primary therapy recommendations by the HT, regardless of the patient's preferences. This was the case for one out of 80 patients in our study cohort.

### Reference model details

The reference model represented an algorithmic emulation of the ESC/EACTS Guidelines for the management of valvular heart disease.^2^ The reference model consisted of a decision tree combined with a weighted sum model (WSM). The decision tree assigned patients to either SAVR or TAVR according to the flowchart outlined in Figure S2. For patients who could not be unambiguously assigned to one or the other treatment modality according to the flowchart, we applied the WSM to arrive at a treatment decision. The WSM linearly combined decision-relevant clinical variables *v* and corresponding weights *w* to arrive at a WSM score *S*:

$$S=\sum_{i} w_{i}v_{i}$$

*S* ≤ 0 indicated a decision for SAVR and a *S* > 0 indicated a decision for TAVR. The input data for the reference model was extracted from the HT protocols. The WSM included variables listed in the ESC/EACTS guidelines^2^ shown in Table S4. The variable weights were determined via a consensus-seeking process among cardiologists and cardiac surgeons (Table S5). Performance metrics were calculated accordingly for the reference model, except that the ICC was set to 1 and the entropy set to 0 due to the purely deterministic nature of the reference model.

**Supplementary Figures**


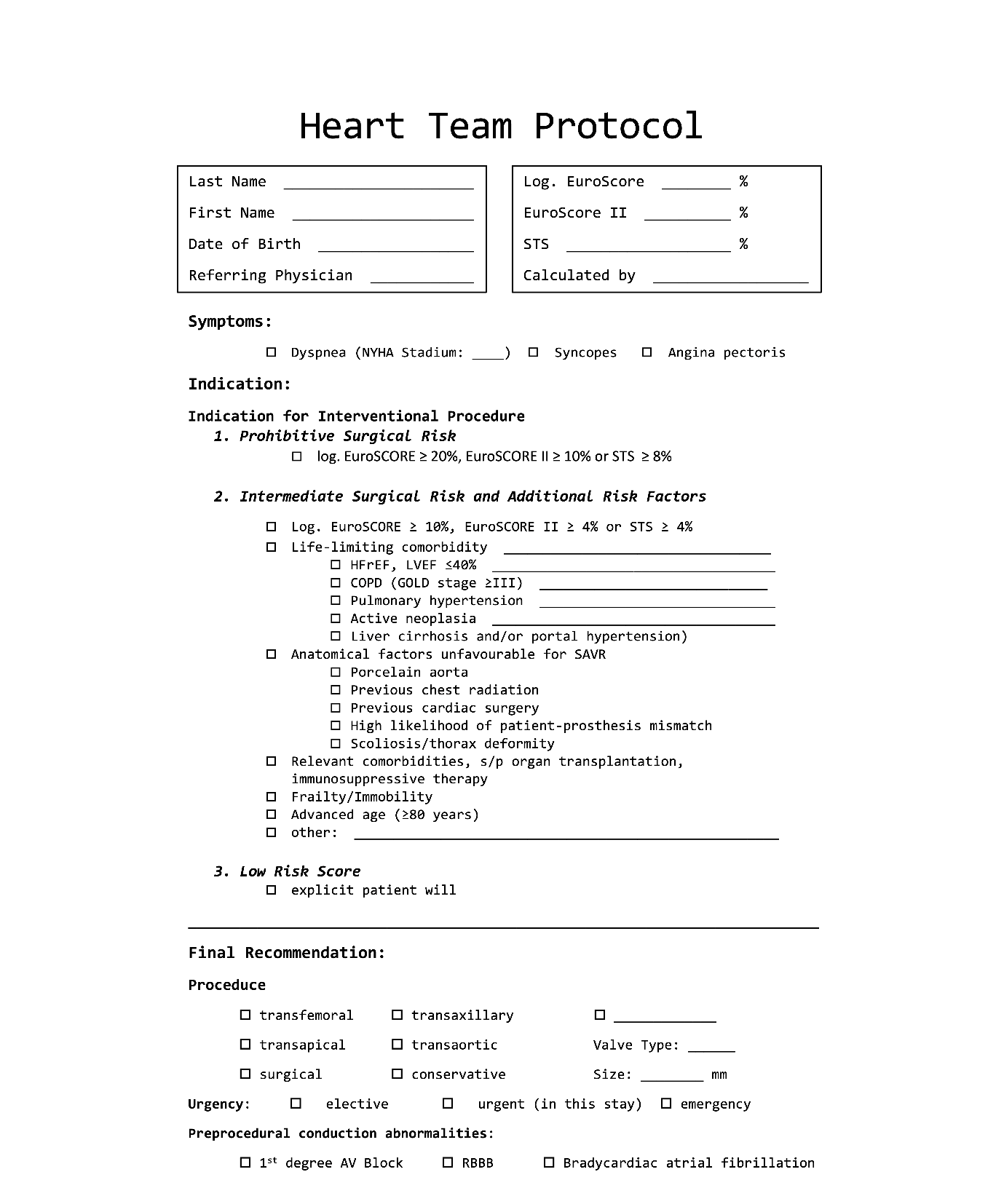


**Figure S1: Heart Team Protocol**

Our institutional Heart Team (HT) protocols included the HT’s treatment decision in addition to decision-relevant patient characteristics. These patient characteristics were used to create case summaries for the SUM and SUM+ experiments and were used as input for the reference model. RBBB: Right bundle branch block. Abbreviations as in Tables S1-2.


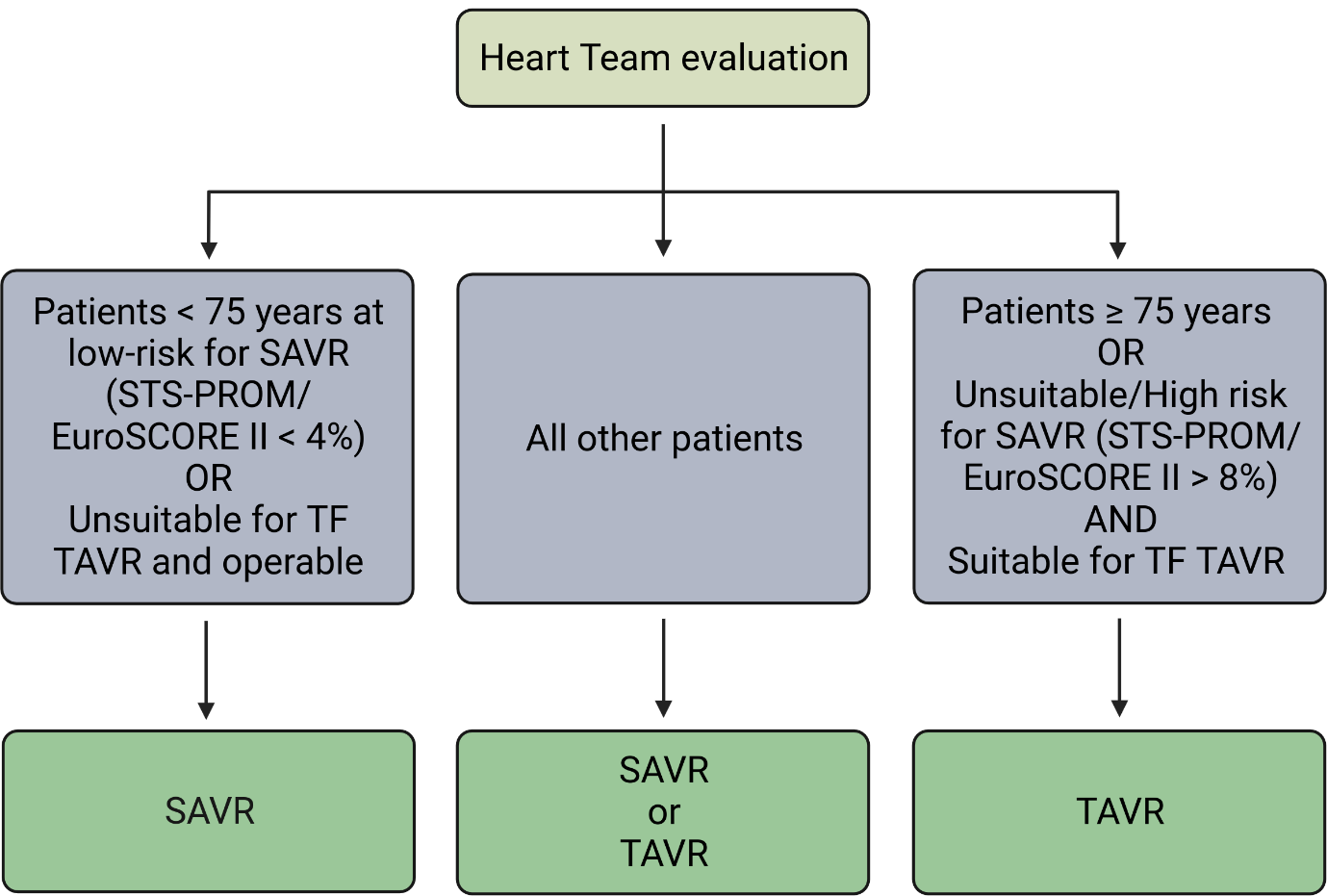


**Figure S2: Flowchart for the management of patients with severe AS**

Management of patients with severe aortic stenosis (AS) according to the 2021 ESC/EACTS Guidelines for the management of valvular heart disease.^2^ TF: Transfemoral. Other abbreviations as in Tables S1-2.

## Supplementary Tables

| **Model Name** | **Version** | **Model Size** (parameters) | **Pre-Training** | **Context Size** (tokens) |
| --- | --- | --- | --- | --- |
| BioGPT | microsoft/BioGPT-Large-PubMedQA | 359 x 10^6^ | large scale biomedical literature | 1,024 |
| ChatGPT-3.5 | gpt-3.5-turbo-0613 | 175 x 10^9^ | 500 x 10^9^ tokens including books, Wikipedia, academic papers, and WordPress-based websites until September 2021. Fine-tuned via reinforcement learning with human feedback (RLHF). | 4,096 |
| ChatGPT-4 | gpt-4-0613 | ~1-1.8 x 10^12^ based on 16 models with 110 billion parameters each, connected by a Mixture of Experts (MoE) | 13 x 10^13^ tokens including data from CommonCrawl, RefinedWeb, and social media until September 2021, with some select information from beyond that date. The model was fine-tuned with data from ScaleAI and internal sources, and RLHF. | 8,192 |
| ChatGPT-4 Turbo | gpt-4-1106-preview | ~1.8 x 10^12^ based on 16 models with ~111 billion parameters each, connected by a Mixture of Experts (MoE) | 13 x 10^13^ tokens including data from CommonCrawl, RefinedWeb, and social media until September 2021, with some select information from beyond that date. The model was fine-tuned with data from ScaleAI and internal sources, and RLHF. | 128,000 |
| ChatGPT-4o | gpt-4o | ~200 x 10^9^ | Multimodal dataset including text, audio, and visual data with text data from publicly available internet data and licensed third-party datasets, and RLHF. | 128,000 |
| Llama 2 | Llama 2-70b-chat | 73 x 10^9^ | 2 x 10^12^ tokens of data from publicly available sources between January and July 2023 SFT on publicly available instruction datasets and RLHF on over one million human-annotated examples. | 4,096 |
| Mistral | mistral-7b-instruct | 7 x 10^9^ | Pre-trainset unknown, fine-tuned using a variety of publicly available conversation datasets. | 4,096 |
| PaLM 2 | text-bison-32k | 540 x 10^9^ | Web documents, books, source code, mathematics, and conversational data | 32,000 |
| DeepSeek-R1 | sonar-reasoning | 671 x 10^9^ MoE system with 21 x 10^9^ parameters per expert | reasoning-specific tasks generated by DeepSeek-V3-Bases, synthetic data, RL with rewards derived from automated checks | 128,000 |

Table S1: Model cards

Model version refers to the identifiers used for each Application Programming Interface (API). Model size is given as the estimated number of trainable parameters. The context size refers to the maximum number of tokens per prompt. Note that detailed model sizes, architectures, training sets and methods are not disclosed by OpenAI and Google and are listed without warranty for correctness. Details on the training data of pre-trained and fine-tuned Llama 2 models are also not publicly available.

| **Model** | **RAW** | **RAW+** | **SUM** | **SUM+** |
| --- | --- | --- | --- | --- |
| BioGPT | 6.4 | 6.6 | 1.0 | 1.0 |
| GPT-3.5 | 2.14 | 2.24 | 1.0 | 1.0 |
| GPT-4 | 1.18 | 1.27 | 1.0 | 1.0 |
| GPT-4 Turbo | 1.0 | 1.0 | 1.0 | 1.0 |
| GPT-4o | 1.0 | 1.0 | 1.0 | 1.0 |
| Llama 2 | 1.85 | 2.0 | 1.0 | 1.0 |
| Mistral | 1.7 | 1.8 | 1.0 | 1.0 |
| PaLM 2 | 1.0 | 1.0 | 1.0 | 1.0 |
| DeepSeek-R1 | 1.0 | 1.0 | 1.0 | 1.0 |

Table S2: Number of prompts per model and experiment

For each model and experiment the average number of prompts needed to present a complete patient case are shown. The number of prompts is contingent upon the context window size (Table S1) and the model-specific tokenizers.

| Experiment | Median Token Count Per Prompt (IQR) |
| --- | --- |
| RAW | 4,445 (2,766 - 6,061) |
| RAW+ | 4,767 (3,088 - 6,383) |
| SUM | 246 (244 - 248) |
| SUM+ | 568 (566 - 570) |

Table S3: Token counts

For each experiment, median token counts per prompt are reported with interquartile ranges (IQR). Abbreviations as in Tables S1-2.

|  | **Favors**  **TAVR** | **Favors**  **SAVR** |
| --- | --- | --- |
| **Clinical characteristics** | | |
| Lower surgical risk | **−** | **+** |
| Higher surgical risk | **+** | **−** |
| Younger age | **−** | **+** |
| Older age | **+** | **−** |
| Previous cardiac surgery (particularly intact coronary artery bypass grafts at risk of injury during repeat sternotomy) | **+** | **−** |
| Severe frailty | **+** | **−** |
| Active or suspected endocarditis | **−** | **+** |
| **Anatomical and procedural factors** | | |
| TAVR feasible via transfemoral approach | **+** | **−** |
| Transfemoral access challenging or impossible and SAVR feasible  Transfemoral access challenging or impossible and SAVR inadvisable | **−**  **+** | **+**  **−** |
| Sequelae of chest radiation | **+** | **−** |
| Porcelain aorta | **+** | **−** |
| High likelihood of severe patient‑prosthesis mismatch (AVA < 0.65 cm^2^/m^2^) | **+** | **−** |
| Severe chest deformation or scoliosis | **+** | **−** |
| Aortic annular dimensions unsuitable for available TAVR devices | **−** | **+** |
| Bicuspid aortic valve | **−** | **+** |
| Valve morphology unfavourable for TAVR (e.g., high risk of coronary obstruction due to low coronary ostia or heavy leaflet/LVOT calcification) | **−** | **+** |
| Thrombus in aorta or LV | **−** | **+** |
| **Concomitant cardiac conditions requiring intervention** | | |
| Significant multi-vessel CAD requiring surgical revascularization | **−** | **+** |
| Severe primary mitral valve disease | **−** | **+** |
| Severe tricuspid valve disease | **−** | **+** |
| Significant dilatation/aneurysm of the aortic root and/or ascending aorta | **−** | **+** |
| Septal hypertrophy requiring myectomy | **−** | **+** |

Table S4: Decision-relevant clinical variables according to the 2021 ESC/EACTS Guidelines for the management of valvular heart disease

Decision‑relevant clinical variables favoring either SAVR or TAVR according to the 2021 ESC/EACTS Guidelines for the management of valvular heart disease.^2^ AVA: aortic valve area, BSA: body surface area, CAD: coronary artery disease, LVOT: Left ventricular outflow tract. Other abbreviations as in Figure S1.

| **Variables from our institutional HT protocol** | **Weights** |
| --- | --- |
| Severe CAD requiring surgical revascularization | -1 |
| Left ventricular ejection fraction < 40 % | 1 |
| COPD | 1 |
| Pulmonary arterial hypertension | 1 |
| Active neoplasia | 1 |
| Liver cirrhosis | 1 |
| Porcelain aorta | 5 |
| Sequelae of chest radiation | 1 |
| Previous cardiac surgery | 1 |
| Expected patient-prosthesis mismatch | 1 |
| Severe chest deformation or scoliosis | 1 |
| Under immunosuppressive therapy | 1 |
| Frailty | 5 |
| Cachexia (BMI < 18.5 kg/m^2^) or morbid obesity (BMI ≥ 40 kg/m^2^) | 1 |

Table S5: Variables of the weighted sum model

Variables and variable weights of the weighted sum model (WSM) are shown. The WSM was applied to arrive at a treatment decision for patients who could not be unambiguously assigned to either surgical- or transcatheter aortic valve replacement according to the flowchart shown in Figure S2. The variable weights were determined via a consensus-seeking process among cardiologists and cardiac surgeons. BMI: body mass index. Other abbreviations as in Table S4.

| **Experiment** | **Prompt Template** |
| --- | --- |
| RAW | PROMPT_INTRO = "Assume a person is with a severe aortic stenosis. {raw_data} Based on the 2021 ESC/EACTS guidelines for the management of valvular heart disease, would the recommended treatment be TAVR or SAVR? Conservative therapy is no option. Please, answer short."  PROMPT_CONT = "Here are more findings for the same patient. {raw_data} Based on the 2021 ESC/EACTS guidelines for the management of valvular heart disease would the recommended treatment be TAVR or SAVR? Conservative therapy is no option. Please, answer short." |
| RAW+ | PROMPT_SUM_CPG = "{cpg_content} {raw_data} Based on the 2021 ESC/EACTS guidelines for the management of valvular heart disease would the recommended treatment be TAVR or SAVR? Conservative therapy is no option. Please, answer short."  PROMPT_CONT = "Here are more findings for the same patient. {raw_data} Based on the 2021 ESC/EACTS guidelines for the management of valvular heart disease would the recommended treatment be TAVR or SAVR? Conservative therapy is no option. Please, answer short." |
| SUM | PROMPT_SUM = "Assume a person is suffering from severe aortic stenosis. {summary} Based on the 2021 ESC/EACTS guidelines for the management of valvular heart disease would the recommended treatment be TAVR or SAVR? Conservative therapy is no option. Please, answer short." |
| cpg_content | "The 2021 ESC/EACTS guidelines for the management of valvular heart disease recommended SAVR in younger patients (<75 years) at low surgical risk (i.e., STS-PROM score < 4 % or EuroSCORE II < 4 %). In older patients (≥75 years), or those who are inoperable or at high surgical risk (i.e., STS-PROM>8 % or EuroSCORE II > 8 %) TAVR is preferred. All other patients should be assessed for either TAVR or SAVR including other factors: For these patients SAVR is favoured: Lower surgical risk (according to STS-score, EuroSCORE II or logistic EuroSCORE), younger age, transfemoral access challenging or impossible and SAVR feasible, aortic annular dimensions unsuitable for available TAVR devices, bicuspid aortic valve, valve morphology unfavourable for TAVR (e.g. high risk of coronary obstruction due to low coronary ostia or heavy leaflet/LVOT calcification), a thrombus within the left ventricle, significant multi-vessel coronary artery disease requiring surgical revascularization, severe primary mitral valve disease, severe tricuspid disease, significant dilatation/aneurysm of the aortic root and/or ascending aorta and septal hypertrophy requiring myectomy. For these patients TAVR is recommended: higher surgical risk (according to STS-score, EuroSCORE II or logistic EuroSCORE), older age, previous cardiac surgery (particularly intact coronary artery bypass grafts at risk of injury during repeat sternotomy), severe frailty, TAVR feasible via transfemoral approach, sequelae of chest radiation, porcelain aorta, high likelihood of severe patient–prosthesis mismatch (AVA <0.65 cm^2^/m^2^ body surface area) and severe chest deformation or scoliosis." |
| summary (Example of a physician-generated case summary) | "The patient is aged 75 years or above*. The logistic EuroSCORE is missing, the EuroSCORE II is 12.99 %, the STS score is missing. The patient does not have coronary heart disease requiring treatment. The patient does not have a left ventricular ejection fraction <= 40 %. The patient does not have chronic obstructive pulmonary disease GOLD Stage III or higher. The patient does not have pulmonary arterial hypertension. The patient has active malignancy. The patient does not have liver cirrhosis. The patient does not have a porcelain aorta. The patient does not have significant thorax deformity or scoliosis. The patient does not have a relevant pre-existing comorbidity that, for example, requires immunosuppression. The patient is not status post chest radiation. The patient is not status post cardiac surgery. The patient is not at high risk for prosthesis‐patient mismatch. The patient is frail. The patient is not suffering from either cachexia or malignant obesity. " *for data protection reasons, the specific age is not given here |

Table S6: Prompt templates

Prompt templates used to communicate with the LLMs. The prompt templates were filled with the original medical reports (RAW) or case summaries (SUM) and/or a resume of the ESC/EACTS guidelines^2^ positionally indicated by the variables raw_data, summary and cpg_content, respectively. Abbreviations as in Table S4.

| **Example Completion** | **Decision** |
| --- | --- |
| "Based on the guidelines, the recommended treatment for a patient with severe aortic stenosis would be TAVR (Transcatheter Aortic Valve Replacement)." | TAVR |
| "Based on the guidelines and the patient's characteristics, the recommended treatment would be TAVR (Transcatheter Aortic Valve Replacement)." |  |
| "Based on the guidelines, the recommended treatment for the patient would be SAVR (surgical aortic valve replacement)." | SAVR |
| "The recommended treatment would be SAVR." |  |
| "Based on the given information, the recommended treatment would be SAVR (Surgical Aortic Valve Replacement)." |  |
| "Based on the findings provided, the recommended treatment would be TAVR (Transcatheter Aortic Valve Replacement) or SAVR (Surgical Aortic Valve Replacement)." | Indeterminate |
| "Based on the guidelines, the recommended treatment for a person with severe aortic stenosis would be TAVR (Transcatheter Aortic Valve Replacement) if they are at higher surgical risk or older, and SAVR" |  |
| "Based on the provided information, it is not possible to determine whether TAVR or SAVR would be the recommended treatment without further details." |  |
| "Based on the guidelines provided, the recommended treatment for a person with severe aortic stenosis would be TAVR (Transcatheter Aortic Valve Replacement) if they are older (≥75 years) or at high surgical risk." |  |

Table S7: LLM completions

Representative LLM responses and their corresponding categorization into a treatment decision. The left column shows the most common responses we retrieved during our experiments.

| **Target** | **Performance metric** | **Description** | **Handling of indeterminate responses** | **Interpretation** |
| --- | --- | --- | --- | --- |
| **Concordance** (“Were the treatment decisions provided by the LLMs concordant with the HT’s treatment decisions?) | **Interrater agreement** | Cohen’s kappa coefficients were used to measure the interrater agreement between treatment decisions provided by the LLMs and the Heart Team. The LLMs’ treatment decisions were aggregated by a majority vote of 10 runs per patient. | Indeterminate responses were classified as "wrong" decisions, meaning they were set to the opposite of the respective HT decision. | Cohen’s kappa coefficients ≤ 0 indicate no agreement, 0.01-0.20 slight, 0.21-0.40 fair, 0.41-0.60 moderate, 0.61-0.80 substantial, and 0.81-1.0 almost perfect agreement.^3^ |
|  | **Accuracy** | Accuracy was defined as the proportion of treatment decisions that agreed with the treatment decisions provided by the Heart Team. | Indeterminate responses were classified as "wrong" decisions, meaning they were set to the opposite of the respective HT decision. | Due to the class imbalance in our patient cohort, a model that exclusively outputs the majority class (i.e., "TAVR") achieves an accuracy of 70 %. |
|  | **Intraclass correlation coefficient (ICC)** | ICCs were used to measure test-retest-reliability given ten runs per patient.  ICCs were calculated based on a one-way random effects, absolute agreement, single-rater model^4^ using Python's pingouin package (version 0.5.3). | Indeterminate responses were re-classified as either "TAVR" or "SAVR" through random sampling with replacement and weights corresponding to the prevalence of HT treatment decisions for TAVR and SAVR in our study cohort. | ICCs < 0.5 indicate poor, 0.50-0.75 moderate, 0.75-0.90 good, > 0.90 excellent test-retest reliability.^4^  ICCs are undefined if the between cluster variance is zero, e.g., if the LLM output was always "TAVR" for every run and every patient for a particular experiment. |
|  | **Entropy** | Shannon entropy^5^ was used to assess the output variation within 10 runs per patient as follows  $H=-\sum p\left( k \right){log}_{2} p(k)$,  with $k\in$ ["TAVR", "SAVR", "indeterminate"] and reported as mean normalized entropy. | Indeterminate responses were included for the calculation of entropy values since entropy allows to quantify output variation for more than two classes. | Entropy values close to 0 indicate no variation in model output. Entropy values close to 1 indicate maximum output variation. |
| **Bias**  ("Were the LLMs’ treatment decisions biased towards TAVR or SAVR?") | **Frequency bias index (FBI)** | FBI^6^ was defined as the ratio of treatment decisions for TAVR given by the LLM and by the Heart Team. | Indeterminate responses were ignored for the calculation of frequency bias indices. | FBI > 1 indicates bias towards TAVR, whereas FBI < 1 indicates bias towards SAVR. |
| **Decidability** ("How often were the LLMs undecided about treatment?") | **Decidability** | Decidability was quantified as the proportion of determinate to indeterminate treatment decisions. | - | - |

Table S8: Performance metrics and imputation strategies

To measure concordance and reliability, we used several metrics. We calculated Cohen’s kappa coefficients to quantify agreement. Accuracy, although intuitive, can be highly misleading in classification tasks with a relevant class imbalance as was the case in our study. Intraclass correlation coefficients (ICCs) were used to measure test-retest reliability based on 10 runs per patient. ICCs are undefined in cases of zero variance and can be skewed by a high proportion of indeterminate answers. To address these limitations, we used Shannon entropy as an additional measure to quantify output variation.

| **Experiment** | **Model** | **Cohen’s Kappa coefficient** | **Frequency Bias Index (FBI)** | **Intraclass Correlation**  **Coefficient (ICC)** | **Entropy** | **Accurate, Indeterminate, Inaccurate** |
| --- | --- | --- | --- | --- | --- | --- |
| **RAW** | BioGPT | -0.18 | 0.95 | 0.73 | 0.32 | (0.32, 0.37, 0.31) |
|  | Mistral | -0.02 | 1.25 | 0.17 | 0.52 | (0.55, 0.2, 0.26) |
|  | Llama 2 | 0.00 | 1.43 | 0.09 | 0.04 | (0.69, 0.01, 0.3) |
|  | PaLM 2 | -0.02 | 1.44 | 1.00 | 0.00 | (0.69, 0.01, 0.3) |
|  | GPT-3.5 | -0.47 | 1.51 | 0.84 | 0.07 | (0.29, 0.54, 0.17) |
|  | GPT-4 | 0.09 | 1.39 | 0.80 | 0.01 | (0.71, 0.02, 0.27) |
|  | GPT-4 Turbo | 0.00 | 1.43 | 0.00 | 0.00 | (0.7, 0.0, 0.3) |
|  | GPT-4o | 0.19 | 1.35 | 0.71 | 0.03 | (0.73, 0.03, 0.24) |
|  | DeepSeek-R1 | 0.22 | 1.30 | 0.19 | 0.17 | (0.75, 0.01, 0.24) |
| **RAW+** | BioGPT | -0.31 | 0.90 | 0.81 | 0.19 | (0.29, 0.41, 0.3) |
|  | Mistral | 0.04 | 1.14 | 0.13 | 0.65 | (0.5, 0.24, 0.25) |
|  | Llama 2 | 0.17 | 1.20 | 0.34 | 0.54 | (0.4, 0.5, 0.1) |
|  | PaLM 2 | -0.02 | 1.44 | 1.00 | 0.00 | (0.69, 0.01, 0.3) |
|  | GPT-3.5 | -0.12 | 1.39 | 0.89 | 0.04 | (0.56, 0.24, 0.2) |
|  | GPT-4 | 0.17 | 1.37 | 0.42 | 0.05 | (0.7, 0.03, 0.27) |
|  | GPT-4 Turbo | 0.00 | 1.43 | - | 0.00 | (0.7, 0.0, 0.3) |
|  | GPT-4o | 0.24 | 1.34 | 0.66 | 0.01 | (0.75, 0.02, 0.23) |
|  | DeepSeek-R1 | 0.40 | 1.19 | 0.48 | 0.17 | (0.8, 0.02, 0.18) |
| **SUM** | BioGPT | 0.04 | 0.82 | 0.27 | 0.46 | (0.29, 0.44, 0.27) |
|  | Mistral | 0.00 | 1.31 | 0.00 | 0.45 | (0.59, 0.12, 0.28) |
|  | Llama 2 | 0.00 | 1.40 | 0.03 | 0.06 | (0.68, 0.0, 0.32) |
|  | PaLM 2 | 0.00 | 1.43 | - | 0.00 | (0.7, 0.0, 0.3) |
|  | GPT-3.5 | -0.25 | 0.90 | 0.85 | 0.12 | (0.41, 0.15, 0.45) |
|  | GPT-4 | 0.44 | 1.15 | 0.69 | 0.12 | (0.81, 0.0, 0.19) |
|  | GPT-4 Turbo | 0.54 | 1.14 | 0.87 | 0.05 | (0.84, 0.0, 0.16) |
|  | GPT-4o | 0.44 | 1.00 | 0.88 | 0.06 | (0.77, 0, 0.23) |
|  | DeepSeek-R1 | 0.34 | 0.66 | 0.44 | 0.34 | (0.65, 0.01, 0.34) |
| **SUM+** | BioGPT | -0.02 | 1.14 | 0.15 | 0.61 | (0.55, 0.18, 0.27) |
|  | Mistral | 0.00 | 1.01 | 0.01 | 0.63 | (0.56, 0.07, 0.36) |
|  | Llama 2 | 0.22 | 1.21 | 0.15 | 0.54 | (0.58, 0.26, 0.16) |
|  | PaLM 2 | 0.47 | 1.23 | 1.00 | 0.00 | (0.81, 0.0, 0.19) |
|  | GPT-3.5 | 0.31 | 0.46 | 0.88 | 0.06 | (0.62, 0.0, 0.38) |
|  | GPT-4 | 0.62 | 0.99 | 0.97 | 0.01 | (0.84, 0.0, 0.16) |
|  | GPT-4 Turbo | 0.61 | 1.12 | 0.93 | 0.03 | (0.86, 0.0, 0.14) |
|  | GPT-4o | 0.58 | 1.12 | 0.97 | 0.01 | (0.84, 0, 0.16) |
|  | DeepSeek-R1 | 0.63 | 0.86 | 0.66 | 0.21 | (0.8, 0.01, 0.19) |
| - | Reference Model | 0.55 | 1.11 | 1 | 0 | (0.83, 0, 0.17) |

Table S9: Performance metrics

Numerical values of the performance metrics portrayed in Figure 2 are shown in addition to the frequencies of accurate, indeterminate and inaccurate treatment recommendations.

## Supplemental References

1. Wolf T, Debut L, Sanh V, et al. Huggingface's transformers: State-of-the-art natural language processing. *arXiv preprint arXiv:191003771*. 2019;

2. Vahanian A, Beyersdorf F, Praz F, et al. 2021 ESC/EACTS Guidelines for the management of valvular heart disease: Developed by the Task Force for the management of valvular heart disease of the European Society of Cardiology (ESC) and the European Association for Cardio-Thoracic Surgery (EACTS). *European Heart Journal*. 2021;43(7):561-632. doi:10.1093/eurheartj/ehab395

3. McHugh ML. Interrater reliability: the kappa statistic. *Biochem Med (Zagreb)*. 2012;22(3):276-82.

4. Koo TK, Li MY. A Guideline of Selecting and Reporting Intraclass Correlation Coefficients for Reliability Research. *J Chiropr Med*. Jun 2016;15(2):155-63. doi:10.1016/j.jcm.2016.02.012

5. Shannon CE. A mathematical theory of communication. *The Bell System Technical Journal*. 1948;27(3):379-423. doi:10.1002/j.1538-7305.1948.tb01338.x

6. Brill KF. A General Analytic Method for Assessing Sensitivity to Bias of Performance Measures for Dichotomous Forecasts. *Weather and Forecasting*. 01 Feb. 2009 2009;24(1):307-318. doi:[10.1175/2008WAF2222144.1](https://doi.org/10.1175/2008WAF2222144.1)
